# Universal Optimization for Non-Clairvoyant Subadditive Joint Replenishment

**DOI:** 10.1007/s00453-026-01389-1

**Published:** 2026-05-12

**Authors:** Tomer Ezra, Stefano Leonardi, Michał Pawłowski, Matteo Russo, Seeun William Umboh

**Affiliations:** 1 Department of Statistics and Operations Research, Tel Aviv University, Tel Aviv, Israel; 2 Sapienza University of Rome, Roma RM, Italy; 3 MIMUW, University of Warsaw and IDEAS NCBR, Warszawa, Poland; 4 School of Computing and Information Systems, The University of Melbourne, Parkville VIC, Australia; 5 ARC Training Centre in Optimisation Technologies, Integrated Methodologies, and Applications (OPTIMA), Melbourne, Australia; 6 Institute of Mathematics, EPFL, Station 8, MA C1 543, Lausanne, 1015 Switzerland

**Keywords:** Joint replenishment problem (JRP), Non-clairvoyant algorithms, Subadditive cost functions, Multi-level aggregation, Online algorithms

## Abstract

The online joint replenishment problem (JRP) is a fundamental problem in the area of online problems with delay. Over the last decade, several works have studied generalizations of JRP with different cost functions for servicing requests. Most prior works on JRP and its generalizations have focused on the clairvoyant setting. Recently, Touitou [44] developed a non-clairvoyant framework that provided an $O(\sqrt{n \log n})$ upper bound for a wide class of generalized JRP, where *n* is the number of request types. We advance the study of non-clairvoyant algorithms by providing a simpler, modular framework that matches the competitive ratio established by Touitou for the same class of generalized JRP. Our key insight is to leverage universal algorithms for Set Cover to approximate arbitrary monotone subadditive functions using a simple class of functions termed *disjoint*. This allows us to reduce the problem to several independent instances of the TCP Acknowledgement problem, for which a simple 2-competitive non-clairvoyant algorithm is known. The modularity of our framework is a major advantage as it allows us to tailor the reduction to specific problems and obtain better competitive ratios. In particular, we obtain tight $O(\sqrt{n})$-competitive algorithms for two significant problems: Multi-Level Aggregation and Weighted Symmetric Subadditive Joint Replenishment. We also show that, in contrast, Touitou’s algorithm is $\Omega (\sqrt{n \log n})$-competitive for both of these problems.

## Introduction

Online problems with delay have received much attention in the last few years. An important family of online problems with delay consists of the Joint Replenishment Problem (JRP) and its variants. A typical instance consists of a sequence of requests that arrive over time. Each request can be one of *n* request types, and the cost of serving a set of requests is a subadditive^1^ function of their types. We assume that the algorithm has oracle access to the service cost function. Requests do not need to be served on arrival but each request accumulates a delay cost while unserved. In particular, each request *q* has an associated delay cost function $d_q$ and its delay cost is $d_q(t)$ if it is served at time *t*. The goal of the problem is to serve all requests while minimizing the total service cost and delay cost. An important special case is the deadline case; this is when requests do not incur delay cost but instead must be served by some given time. We call this family of problems *Subadditive JRP*, which is a strict generalization of the classic JRP.

These problems can be studied under the clairvoyant and non-clairvoyant settings. In the *clairvoyant* setting, when a request *q* arrives, the algorithm is given the entire delay cost function $d_q$ (or its deadline in the case of deadlines). In contrast, in the *non-clairvoyant* setting, the algorithm only knows of the delay cost accumulated so far. In the case of deadlines, the algorithm only knows whether the request’s deadline is now (and must be served immediately) or later.

Most previous works on Subadditive JRP have focused on the clairvoyant setting. Key problems within the family of Subadditive JRP include (in increasing order of generality): TCP Acknowledgement [15, 25, 37], Joint Replenishment Problem [12, 16, 20, 22], and Multi-Level Aggregation (MLA) [7, 9, 10, 14, 42]. For general subadditive service cost functions, deterministic O(logN) (where *N* is the number of requests) and O(logn) upper bounds are known ( [23] and [8], respectively).

There is much less work in the non-clairvoyant setting. For a small number of problems, such as TCP Acknowledgement and Set Cover with Delay [3], clairvoyance is not required in the sense that the same competitive ratio can be attained in both the clairvoyant and non-clairvoyant settings. However, Azar et al. [5]’s lower bound for Online Service with Delay (a different family of online problems with delay) can be translated into an $\Omega (\sqrt{n})$ lower bound against deterministic algorithms for JRP, and thus, MLA and Subadditive JRP. In contrast, clairvoyant Subadditive JRP has a O(logn) competitive ratio [8]. Recently, Le et al. [41] showed that randomization does not help in breaking the $\Omega (\sqrt{n})$ barrier and also developed algorithms for JRP and MLA with matching and nearly-matching upper bounds. Shortly after, Touitou [44] presented a general non-clairvoyant framework for Subadditive JRP with a deterministic $O(\sqrt{n \log n})$ competitive ratio.

### Our Results

**Fig. 1:**
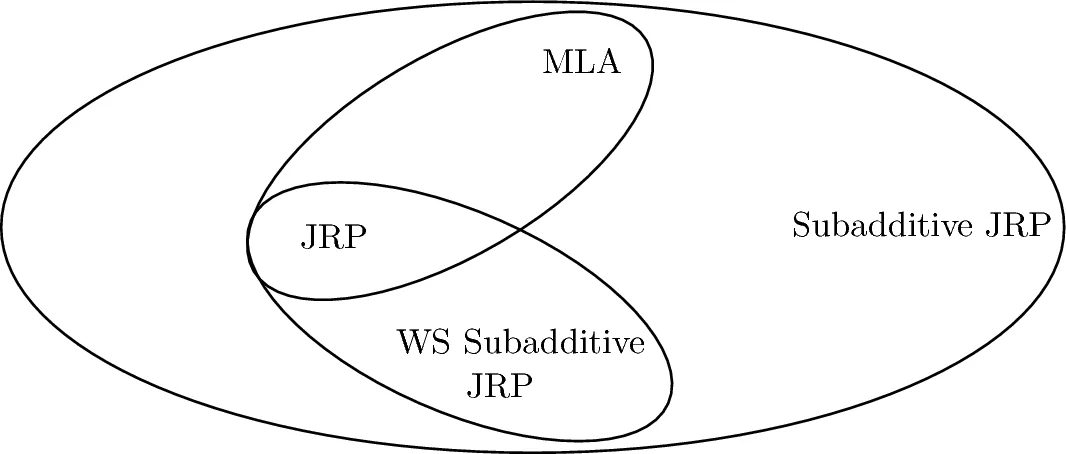
The hierarchy of problems we consider: ${\text {JRP}} \subseteq {\text {MLA}}, {\text {WS Subadditive JRP}}\subseteq {\text {Subadditive JRP}}$, where “WS” is an abbreviation for “Weighted Symmetric”.

Our main contribution is a simple, modular framework for non-clairvoyant Subadditive JRP that matches the current-best competitive ratio of $O(\sqrt{n \log n})$, and yields tight $O(\sqrt{n})$ competitive ratios for the key problems of Multi-Level Aggregation and Weighted Symmetric Subadditive Joint Replenishment (see formal definitions in Section 1.1.2). We also show that the framework of Touitou [44] is $\Omega (\sqrt{n \log n})$ for these problems. We now formally define these problems and state our results (please refer to Figure 1 for an illustration).

#### General Framework for Subadditive JRP

**Subadditive JRP.** We have a set *U* of *n* request types and a monotone non-decreasing, subadditive *service function* $f: 2^U \mapsto \mathbb {R}_{\ge 0}$ that satisfies f(∅)=0. Requests arrive over time. Each request *q* has a type $h_q \in U$, an arrival time $a_q$, and a non-decreasing, continuous delay function $d_q$. We also assume that $\lim _{t \rightarrow \infty } d_{q}(t) = \infty$ which ensures that all requests will be served eventually.^2^ At any point in time, the algorithm can serve a subset *Q* of the requests that have arrived and incur a service cost of $f(S_Q)$ where $S_Q = \{h_q: q \in Q\}$ is the set of types of *Q*. Let $C_q$ be the time when request *q* was served. The *delay cost* of request *q* is $d_q(C_q)$.^3^ The goal is to serve all requests while minimizing the sum of the total service and delay costs.

**Approximating set functions.** The core idea underlying our framework is the following simple but powerful observation. Given two set functions *f*, *g* over the same ground set *U* of *n* elements, we say that *g* is an α*-approximation* of *f* if $f(S) \le g(S) \le \alpha f(S)$ for every S⊆U. Our observation is that for a given subadditive service function *f*, if we can α-approximate *f* by a simpler service function *g*, then we can reduce any instance of Subadditive JRP with service function *f* to one with *g* instead. In fact, this leads us to the following simplification of the problem.

**Disjoint TCP Acknowledgement.** In Disjoint TCP Acknowledgement, we have a set *U* of *n* request types. We also have a partition of *U* into subsets $S_1, \ldots , S_k$ with costs $c_1, \ldots , c_k$. For a subset S⊆U, we have $f(S) = \sum _{i = 1}^k c_i \cdot 1\!\!1\left\{ S_i \cap S \ne \emptyset \right\}$. In other words, we pay $c_i$ for every part $S_i$ that intersects with *S*. Such a function is called a *disjoint service function*. Observe that when k=1, this is equivalent to the TCP Acknowledgement problem; when k>1, this corresponds to several independent instances of TCP Acknowledgement. The 2-competitive algorithm for TCP Acknowledgement of [25] can be easily extended to a 2-competitive algorithm for Disjoint TCP Acknowledgement (see Section 2.1).

We now state our main technical lemma.

##### Lemma 1.1

(Reduction Lemma) If there exists a disjoint service function *g* that α-approximates *f*, then there exists a non-clairvoyant algorithm that is 2α-competitive non-clairvoyant algorithm for every Subadditive JRP instance with service cost function *f*.

A major advantage of our Reduction Lemma is that it reduces the task of designing and analyzing an online algorithm for a Subadditive JRP problem to the much cleaner task of showing that the corresponding service function *f* can be approximated by a disjoint service function well. In particular, this boils down to finding a partition of the set of request types *U* into subsets $S_1, \ldots , S_k$, for some *k*, such that the following quantity is small

$$\begin{aligned} \max _{S \subseteq U} \frac{\sum _{i = 1}^k f(S_i) \cdot 1\!\!1\left\{ S_i \cap S \ne \emptyset \right\} }{f(S)}. \end{aligned}$$

For general Subadditive JRP, our key insight is that the problem of approximating an arbitrary service function *f* by a disjoint service function can be reformulated as the Universal Set Cover problem.

**Universal Set Cover (USC).** An instance of the Universal Set Cover (USC) problem consists of a universe *U* of *n* elements, a collection $\mathcal {C}$ of subsets of *U*, and costs *c*(*S*) for each set $S \in \mathcal {C}$. A solution is an assignment *a* of each element *e* to a set $a(e) \in \mathcal {C}$. For any subset X⊆U, define $a(X) = \{a(e): e \in X\}$. The *stretch* of the assignment *a* is $\max _{X \subseteq U} c(a(X)) / OPT (X)$ where OPT(X) is the cost of the optimal set cover of *X*.

Jia et al. [35] introduced the Universal Set Cover problem and showed that a $O(\sqrt{n \log n})$-stretch assignment can always be found efficiently. We show that this implies that any subadditive service function *f* can be approximated by a disjoint service function to within a factor of $O(\sqrt{n \log n})$ (Lemma 2.2). Together with our Reduction Lemma, we get a deterministic $O(\sqrt{n \log n})$-competitive algorithm for Non-Clairvoyant Subadditive JRP, matching the current state-of-the-art [44].

#### MLA and Weighted Symmetric Subadditive JRP

One main technical contribution of the paper is to exploit the inherent structure of the MLA and Weighted Symmetric Subadditive JRP functions to show that they can be $O(\sqrt{n})$-approximated by disjoint service functions. We then employ the Reduction Lemma to prove tight $O(\sqrt{n})$-competitive ratios for the two corresponding problems.

**Multi-Level Aggregation.** In the Multi-Level Aggregation (MLA) problem, the service function *f* is defined by a rooted *aggregation tree* *T*, where each node corresponds to a different request type. Let *r* be the root of *T* and let *c*(*v*) be the cost of node *v* for each v∈T. For a subset *V* of nodes, *f*(*V*) is defined to be the total cost of the nodes in the minimal subtree connecting *V* to *r*.

##### Theorem 1.2

There exists an efficient deterministic $O(\sqrt{n})$-competitive algorithm for the Non-Clairvoyant Multi-Level Aggregation problem.

To show the above result, given Lemma 1.1, our goal is to find a good partition *P* of the tree *T*’s nodes into subtrees and subforests (that we refer to as clusters). More precisely, let us use *P* to define a disjoint service function *g* where for each subset *V* of nodes of *T*, $g(V) = \sum _{C \in P: C \cap V \ne \emptyset } f(C)$.

The crucial idea is to notice that since we aim for the gap of order at most $\sqrt{n}$ between *g* and *f*, we can see it as *g* being assigned a budget of roughly $\sqrt{n}f(V)$ to serve *V* for each subset *V* of *T*’s nodes. Since the cost that *f* incurred on a set *V* equals the cost of the minimal subtree connecting all the nodes in *V* to the root *r* of *T*, the value of *g*(*V*) cannot exceed $\beta \sqrt{n}$ times this cost for some fixed $\beta \in \mathbb {N}$. To achieve this, we generate a partition consisting of two types of clusters. First are the subtrees rooted at “expensive” nodes. The intuition is that their cost alone multiplied by $\alpha \sqrt{n}$ for some $\alpha \in \mathbb {N}$ is enough to “cover” the cost of both their subtree and the path to *r*. The second type is the clusters that contain more than $\sqrt{n}$ nodes, since there cannot be many of them.

**Weighted Symmetric Subadditive JRP.** In Weighted Symmetric Subadditive JRP, the service function *f* is a function of the total weight $w(S) = \sum _{i \in S} w_i$ of the set of types being served. In particular, *f* is a monotone non-decreasing subadditive function with f(S)=f(w(S)) and f(0)=0, that satisfies that for every weights *x*, *y*, f(x+y)≤f(x)+f(y). We refer to these functions as *weighted symmetric subadditive*.

##### Theorem 1.3

There exists an efficient deterministic $O(\sqrt{n})$-competitive algorithm for the Non-Clairvoyant Weighted Symmetric Subadditive Joint Replenishment problem.

As in the MLA case, given Lemma 1.1, our goal is to devise a partitioning algorithm inducing a disjoint service function that $O(\sqrt{n})$-approximates the corresponding weighted symmetric subadditive service cost function. We first consider the special case where each weight equals 1. In this scenario, the service function *f* is symmetric and becomes a function of the cardinality of the set of types being served. Consequently, the partition of elements should ideally reflect this symmetry by ensuring equal-sized parts. Determining the optimal part size balances the subadditivity benefits of larger sizes against increased costs for smaller sets. In the weighted case, elements are grouped by size, but this can create too many sets. To address this, light-weight elements are partitioned first, followed by heavier elements split by a factor of two when feasible for finer granularity.

#### Running time of Algorithms and Reductions

Regarding the running time of our algorithms, we stress that, in the case of Multi-Level Aggregation and Weighted Symmetric Subadditive JRP, the reductions are executed in polynomial time. However, the reduction for general subadditive functions is executed in exponential time, as we need to create a set for each subset of types.

#### Lower bounds on approximating subadditive service functions.

Since Non-Clairvoyant MLA and Weighted Symmetric Subadditive JRP have a $\Omega (\sqrt{n})$ lower bound [5, 41], the Reduction Lemma implies that MLA and Weighted Symmetric Subadditive JRP service functions do not admit $o(\sqrt{n})$-approximation by disjoint service functions. Nevertheless, we also give direct proofs in Sections 3 and 4, respectively. The latter provides a simpler alternative proof for the $\Omega (\sqrt{n})$ lower bound for unweighted Universal Set Cover shown in [35]. We also show, in Proposition 5.1, that Jia et al.’s analysis of their Universal Set Cover algorithm [35] is tight. Thus, we need a different approach to $o(\sqrt{n \log n})$-approximate arbitrary subadditive service functions by disjoint service functions. Finally, in Proposition 5.2, we exhibit an MLA and Weighted Symmetric Subadditive JRP instance where Touitou’s algorithm [44] can only achieve an $\Omega (\sqrt{n \log n})$-approximation to the respective service cost functions.

### Future Directions

Our work leaves several tantalizing open questions. The main open problem is whether subadditive service functions admit better than $O(\sqrt{n \log n})$-approximation by disjoint service functions. This would immediately improve the competitive ratio for general non-clairvoyant Subadditive JRP. It would also be interesting to find better approximations of other interesting subclasses such as XOS and submodular functions.

### Further Related Work

**Network Design with Delay.** Network Design with Delay is very closely related to Subadditive JRP. In Network Design with Delay, we are given a universe of *n* request types and *m* items with costs. Each request type *h* has a corresponding upwards-closed collection $\mathcal {C}_h$ of subsets of items that satisfy it. At any point in time, the algorithm can transmit a set of items. A request of type *h* is served by a transmission that contains some subset in $\mathcal {C}_h$. Some specific problems are Set Cover with Delay [3, 23, 43], Facility Location [7, 8, 11] and other network design problems [7, 8]. Network Design with Delay is equivalent to Subadditive JRP as the optimal cost of satisfying a subset of request types is subadditive, and Subadditive JRP can be formulated as Set Cover with Delay with exponentially many sets. The difference between Network Design with Delay and Subadditive JRP is that in Subadditive JRP we are given a direct oracle access to the optimal cost of serving sets of requests. This allows for designing efficient algorithms for Subadditive JRP.

**Online problems with delay.** There has been a lot of work on other online problems with delay as well. In Online Service with Delay, we are given one or multiple servers on a metric space. Requests arrive on points of the metric space and are served when a server is moved to their location. In Online Matching with Delay, we are given an underlying metric space. Requests arrive on points of the metric space and are served when they are matched. Most of the work on Online Service with Delay [5, 19, 32, 33, 38, 45] and Online Matching with Delay [1, 2, 4, 6, 17, 18, 27, 29, 30, 40] has been in the clairvoyant setting. Nevertheless, non-clairvoyant algorithms have been designed for Online Service with Delay [38] and Online Matching with Delay [27].

**Approximating subadditive functions.** The approximation of subadditive functions has been a focal point of research, at least since the introduction of the complement-free hierarchy of functions introduced in [39]. This consists of the class of submodular function, which is strictly contained into the XOS class, which in turn is strictly contained in the general subadditive class.^4^ As for approximation, it is known that XOS approximates subadditive within a factor of O(log(n)), which is tight [21, 26]. The approximability gap between Submodular and XOS is $\Theta (\sqrt{n})$ [13, 31]. In a similar vein, [24] prove that Gross-Substitute functions (first introduced in [36]) cannot approximate submodular set functions within a factor better than $\Omega \left( \frac{\log (n)}{\log \log (n)}\right)$. In the context of *symmetric* function approximation, [28] show that symmetric subadditive, symmetric XOS and symmetric submodular^5^ functions are all 2-close to each other, which is tight.

## Subadditive Joint Replenishment

In this section, we prove our Reduction Lemma (Lemma 1.1) and apply it to Subadditive JRP.

### Reduction Lemma

We begin by showing that there is a simple deterministic 2-competitive algorithm for Disjoint TCP Acknowledgement via a straightforward extension of the classic algorithm for TCP Acknowledgement of [25].

In the following, we use λ to denote a service and $Q_\lambda$ to be the set of request types transmitted by λ. We also use OPT to mean both the optimal solution and the cost of the optimal solution.

#### Lemma 2.1

There is a deterministic 2-competitive algorithm for Disjoint TCP Acknowledgement.

#### Proof

Suppose there is a partition of *U* into subsets $S_1, \ldots , S_k$ with costs $c_1, \ldots , c_k$ and $f(S) = \sum _{i = 1}^k c_i \cdot 1\!\!1\left\{ S_i \cap S \ne \emptyset \right\}$. Our algorithm works as follows: for each set $S_i$, transmit $S_i$ whenever the pending requests in $S_i$ have accumulated a total delay equal to $c_i$. There is always such a point by the Intermediate-Value Theorem, since the delay cost is continuous, non-decreasing, and goes to infinity (as $t \rightarrow \infty$).

It is clear that the total service cost of the algorithm is equal to its total delay cost. We now show that the latter is at most the cost of the optimal solution. To this end, let us consider the cost of the optimal solution. Suppose that the optimal solution makes a set of services $\Lambda ^*$. Let $\Lambda ^*_i$ denote the subset of services that transmit a request type in $S_i$. The total service cost of the optimal solution is then

$$\begin{aligned} \sum _{\lambda \in \Lambda ^*} f(Q_\lambda ) = \sum _{\lambda \in \Lambda ^*} \sum _{i=1}^k c_i \cdot 1\!\!1\left\{ S_i \cap Q_\lambda \ne \emptyset \right\} = \sum _{i=1}^k c_i \cdot |\Lambda ^*_i|. \end{aligned}$$

Define $d^OPT _q$ and $d^ALG _q$ to be the delay cost of *q* in the optimal solution and algorithm’s solution, respectively. Let $OPT _i = c_i \cdot |\Lambda ^*_i| + \sum _{q: h_q \in S_i} d^OPT _q$. This is the total cost that OPT incurs on requests on $S_i$. Observe that $OPT = \sum _{i=1}^k OPT _i$.

We now show that $\sum _{q: h_q \in S_i} d^ALG _q \le OPT _i$ for each set $S_i$. Suppose that the algorithm transmits $S_i$ at times $t_1, \ldots , t_\ell$. Since every request must be served eventually, no request with type in $S_i$ arrives after $t_\ell$. Consider the intervals $[0, t_1], (t_1, t_2], \ldots (t_{\ell -1}, t_\ell )$. By construction, the delay cost of the algorithm is exactly $\ell c_i$. For each interval *I*, let *Q*(*I*) denote the requests with types in $S_i$ that arrived during the interval. During *I*, the optimal solution either transmits a type in $S_i$ or incurs a delay cost of $c_i$ on the requests in *Q*(*I*). Since the intervals are disjoint, $OPT _i \ge \ell c_i$, as desired.

The lemma now follows from the fact that the total service cost of the algorithm is exactly its delay cost, which in turn is at most OPT.□

We are now ready to prove the Reduction Lemma which we restate here.

**Lemma 1.1**(Reduction Lemma) *If there exists a disjoint service function* *g* *that* α*-approximates* *f*, *then there exists a non-clairvoyant algorithm that is* 2α-*competitive non-clairvoyant algorithm for every Subadditive JRP instance with service cost function* *f*.

#### Proof

Lemma 2.1 implies that it suffices to reduce the Subadditive JRP instance to an instance of Disjoint TCP Acknowledgement losing at most a factor of α. Let *Q* be the set of requests of the Subadditive JRP instance and let $OPT ^f$ denote the cost of the optimal solution. Our reduction creates an instance of Disjoint TCP Acknowledgement with the same set of requests but with service cost function *g*. Let $OPT ^g$ denote the cost of the optimal solution to the instance of Disjoint TCP Acknowledgement. We now show that $OPT ^f \le OPT ^g \le \alpha OPT ^f$. Let Λ be a feasible solution to *Q*, $c^f(\Lambda )$ be its cost in the Subadditive JRP instance and $c^g(\Lambda )$ be its cost in the Disjoint TCP Acknowledgement instance. The delay cost of Λ is the same in both instances. The service cost of Λ in the Subadditive JRP instance has cost $\sum _{\lambda \in \Lambda } f(Q_\lambda )$ and in the Disjoint TCP Acknowledgement instance, it has cost $\sum _{\lambda \in \Lambda } g(Q_\lambda )$. Since *g* α-approximates *f*, we get that $c^f(\Lambda ) \le c^g(\Lambda ) \le \alpha c^f(\Lambda )$. This implies that $OPT ^f \le OPT ^g \le \alpha OPT ^f$, as desired. □

### Applying the Reduction Lemma to Subadditive JRP

We use the Reduction Lemma proved earlier to give a simple deterministic $O(\sqrt{n \log n})$-competitive algorithm for Non-Clairvoyant Subadditive JRP. The main insight is to reduce the problem of showing that an arbitrary service function *f* can be approximated by a disjoint service function to the Universal Set Cover problem.

#### Lemma 2.2

Suppose every instance of USC admits an α-stretch assignment. Then every subadditive service function *f* can be α-approximated by some disjoint service function *g*.

#### Proof

We will construct an instance of USC and use the α-stretch assignment to construct *g*. Consider the instance of USC with universe *U*, $\mathcal {C}= 2^{U}$, and c(S)=f(S) for every $S \in \mathcal {C}$. Note that OPT(S)=f(S) since *f* is monotone non-decreasing and subadditive.

Let *a* be an α-stretch assignment for this USC instance. Suppose $a(U) = \{S_1, \ldots , S_k\}$. Since *a* maps each element to a set containing it, we have that $a^{-1}(S_i) \subseteq S_i$. Define a new assignment $a^\prime$ as the function that maps elements in the preimage of $S_i$ under *a* to the preimage itself. For instance, if $a^{-1}(S_i) \subsetneq S_i$ then for all $x \in a^{-1}(S_i)$ we have $a^\prime (x) = a^{-1}(S_i)$. Thus, $a^\prime (U) = \{S^\prime _1, \ldots , S^\prime _l\}$ are disjoint and partition *H*. Moreover, since *f* is monotone non-decreasing, $a^\prime$ too has α-stretch because its cost is at most that of *a*, i.e., $c(a^\prime (S)) \le c(a(S))$ given that $a^\prime (S) \subseteq a(S)$. Define the disjoint service function *g* with the partition $\{S^\prime _1, \ldots , S^\prime _l\}$ and costs $c_1, \ldots , c_l$ where $c_i = f(S_i)$. Observe that $g(S) = c(a^\prime (S)) \ge OPT (S) = f(S)$. Since $a^\prime$ has α-stretch, we get that for every *S*, $f(S) \le g(S) \le \alpha f(S)$. □

Jia et al. [35] showed that every instance of USC has a $O(\sqrt{n \log n})$-stretch assignment. Together with the above lemma, we get the following theorem.

#### Theorem 2.3

For every subadditive service function *f*, there is a disjoint service function *g* that $O(\sqrt{n \log n})$-approximates *f*.

Combining this with the Reduction Lemma yields the desired theorem.

#### Theorem 2.4

There is a deterministic $O(\sqrt{n \log n})$-competitive algorithm for Non-Clairvoyant Subadditive JRP.

## Multi-Level Aggregation

In this section, we consider the Multi-Level Aggregation (MLA) problem. Let T=(U,E) be a rooted tree defined over the universe *U* of *n* request types and let $c: U \mapsto \mathbb {R}_{\ge 0}$ be a cost function assigning weights to the nodes. We recall that *c* determines the service function $f: 2^{U} \mapsto \mathbb {R}_{\ge 0}$ for this problem as *f* assigns to each subset of nodes V⊆U the cost of the minimal subtree that connects all the nodes in *V* to the root *r*. Here, we prove that for every MLA service function *f*, there exists a disjoint service function $g: 2^{U} \mapsto \mathbb {R}_{\ge 0}$ that $O(\sqrt{n})$-approximates *f*. In other words, we show that for every MLA instance (*T*, *c*), there exists a partition $P_1, \ldots , P_k$ of nodes of *T* for some *k* (which defines $g(X)= \sum _{i\in [k]} f(P_i) \cdot 1\!\!1\left\{ P_i \cap X \ne \emptyset \right\}$ for all X⊆U), such that for all V⊆U, it holds that $g(V)/f(V) \le O(\sqrt{n})$. Moreover, one can find such a partition in polynomial time.

### Notation and Algorithm Overview

Throughout this section, we assume tree *T* is the current MLA instance that we work with and thus is known from the context. In what follows, we refer to the *maximal subtree* of *T* rooted at node *v* and to the *path* connecting *v* to the root *r* by simply writing *T*(*v*) and *R*(*v*), respectively. Moreover, to denote these objects with node *v* excluded, we use $T_{o}(v)$ and $R_{o}(v)$. Finally, we let *C*(*v*) be the set of *v*’s *children* in *T*.

**Fig. 2:**
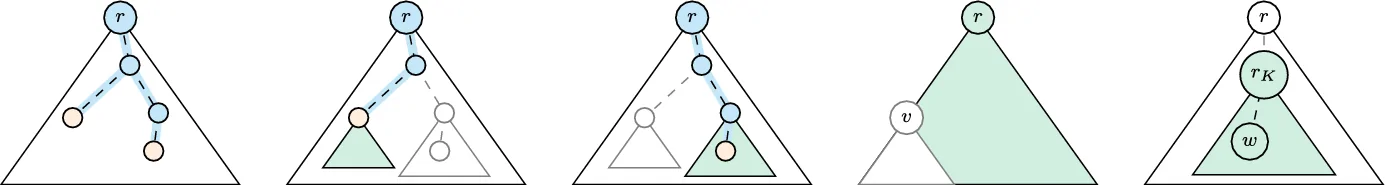
In the first three figures we show the costs of serving the orange nodes. The first figure corresponds to the cost of *f* on these nodes. The following two figures show the cost of *g* on these nodes, assuming that they belong to different clusters *A* and *B*. Fourth figure shows the set of active nodes in the tree (colored in green) after *T*(*v*) gets clustered. Fifth figure presents the setting in Proposition 3.7.

First, we present the idea behind our approach. Recall that our goal is to find a partition $\mathcal {P} = \{P_1, \ldots , P_k\}$ of nodes of *T* such that the gap between the values *f*(*V*) and *g*(*V*) is at most of order $\sqrt{n}$ for all the sets V⊆U. Here, *f*(*V*) is the cost of minimal subtree $T_V$ of *T* that connects all the nodes in *V* to the root, as stated before. On the other hand, *g*(*V*) needs to cover the costs of all the parts in $\mathcal {P}$ that intersect with *V*. For instance, if *V* intersects exactly two parts *A* and *B* in $\mathcal {P}$, then g(V)=f(A)+f(B). Although these parts themselves are disjoint by the definition of partition $\mathcal {P}$, as we pay for each of them separately in *g* (by paying for set *A*, we mean generating the cost of *f*(*A*)), we incur not only the costs of their nodes *c*(*A*) and *c*(*B*) but also the costs of the paths that connect them to the root *r* (see Figure 2).

Note that this process can cause us to incur two types of additional costs with respect to the optimal value *f*(*V*). First, both parts *A* and *B* may contain not only the nodes in *V* but also their neighbours, for which we need to pay as well. Second, as we pay for each part separately, we may be forced to pay for some nodes on the paths to the root multiple times (see Figure 2).

Since *f*(*V*) is equal to the cost of the nodes in $T_V$ and we aim for *g* to be $\sqrt{n}$-approximation of *f*, the intuition is that *g* can afford to pay the cost of each node in $T_V$ roughly $\sqrt{n}$ times (as this gives the desired ratio). This observation provides the foundations for our algorithm. Let us remark that at the beginning, all the nodes in *T* are unpartitioned, i.e., $\mathcal {P} = \emptyset$. Our algorithm revolves around two procedures. The first one can be seen as assigning each node *v* in *T* a budget of $\alpha \sqrt{n} \cdot c(v)$ for some $\alpha \in \mathbb {N}$. A vertex *v* may then use such a budget to create a new part *K* in the partition. To determine whether this is possible, we check that the total cost of all unpartitioned nodes in *T*(*v*) and the cost of *R*(*v*) both fall within *v*’s budget. If this is the case, we add *K* to $\mathcal {P}$, calling the node *v* *heavy* and the part *K* a *heavy cluster*.

Whenever the first procedure cannot be applied, i.e., there are no vertices that can generate heavy clusters from the unpartitioned nodes, we run the second procedure. The idea then is to find a subtree (or a family of subtrees) of size roughly $\sqrt{n}$ (details to be presented later) and group them together into a new part in the partition. We call this part a *light cluster*. If some nodes become heavy after this action (as their descendants got clustered), we go back to the first procedure, which starts a new iteration of the main algorithm. From this point on, we use the term cluster for the parts of the partition, as we subsequently distinguish them as either heavy or light.

Notice that the idea behind the second procedure is to upper bound the number of times we need to pay the cost of the paths connecting the clusters to the root *r*. Since *T* has *n* nodes and each light cluster is of size close to $\sqrt{n}$, we can only create roughly $\sqrt{n}$ such clusters. Thus, even when *V* intersects all the light clusters, we pay for the nodes in $T_V$ at most $O(\sqrt{n})$ times, which we can afford. It remains to estimate the cluster costs, which follow in the next section.

### MLA Partitioning Algorithm

**Heavy clusters.** Let us first present two definitions. Here, we assume that whenever we are given a partially created partition $\tilde{\mathcal {P}}$ of nodes of an MLA tree *T*, then the set of nodes it already partitioned, i.e., $V(\tilde{\mathcal {P}}) = \bigcup \tilde{\mathcal {P}}$, does not disconnect *T*, i.e., tree $T' = T \setminus \bigcup \tilde{\mathcal {P}}$ is a subtree of *T*.

#### Definition 3.1

Let *T* be a tree given in an MLA instance, denote the set of its nodes by *U*, and let $\tilde{\mathcal {P}}$ be a partially created partition of *U* (i.e., $V(\tilde{\mathcal {P}}) = \bigcup \tilde{\mathcal {P}}$ is a proper subset of *U*). Then we call all the nodes that are not partitioned yet, i.e., belong to $U \setminus V(\tilde{\mathcal {P}})$, *active* (see Figure 2). We use the notation of $V_{act}$ to restrict any subset *V* of nodes in *T* to the nodes that are active.

#### Definition 3.2

Let (*T*, *c*) be an MLA instance, and let $\tilde{\mathcal {P}}$ be a partially created partition of *T*’s nodes. We say that an active node *v* is *heavy* if the costs of path *R*(*v*) and subtree $T_{act}(v)$ are at most $4\sqrt{n} \cdot c(v)$ each. If we extend $\tilde{\mathcal {P}}$ by adding $T_{act}(v)$, we call this new part a *heavy* cluster. We refer to *v* as the root of this heavy cluster.

Now, we can prove a simple fact about heavy clusters.

#### Proposition 3.3

Let (*T*, *c*) be an MLA instance. Take any partition $\mathcal {P}$ of nodes of *T* and let $P_{h,1}$, …, $P_{h,s}$ be any sublist of its heavy clusters in $\mathcal {P}$. We denote their roots by $v_{h,1}$, …, $v_{h,s}$, respectively, and the set containing them by $V_h$. Then, it holds that $\sum _{i=1}^s f(P_{h,i}) \le 8\sqrt{n} \cdot f(V_h)$.

#### Proof

By Definition 3.2, we have that for each node $v_{h,i}$ the following is satisfied: $c(P_{h,i}) \le 4\sqrt{n} \cdot c(v_{h,i})$ and $c(R(v_{h,i})) \le 4\sqrt{n} \cdot c(v_{h,i})$. The first inequality here comes from the fact that cluster $P_{h,i}$ was the set of all active nodes (Definition 3.1) contained in the subtree $T(v_{h,i})$ at the moment it was created (i.e., it was $T_{act}(v_{h,i}))$. Hence, it holds that

1 $$\begin{aligned} \begin{aligned} f(P_{h,i})&= c(P_{h,i}) + c(R_{o}(v)) \le c(P_{h,i}) + c(R(v)) \\&\le 4\sqrt{n} \cdot c(v_{h,i}) + 4\sqrt{n} \cdot c(v_{h,i}) = 8\sqrt{n} \cdot c(v_{h,i}), \end{aligned} \end{aligned}$$

where the first equality comes from the fact that $P_{h,i}$ is a subtree, which means that the minimal tree containing all its nodes and the root *r* is only missing the path from *v* to *r* (with *v* excluded as we already counted it in the cluster). Moreover, let us notice that $f(V_h) \ge \sum _{i=1}^s c(v_{h,i})$, as it is the cost of the minimal tree containing all the nodes $v_{h,i}$. Thus, to obtain the desired inequality, we only need to sum (1) over all the heavy clusters $P_{h,i}$ and then apply the inequality above. □

**Light clusters.** Here, we present a procedure that generates a light cluster.

#### Definition 3.4

Let (*T*, *c*) be an MLA instance, and let $\tilde{\mathcal {P}}$ be a partially created partition of *T*’s nodes. We say that a subset *K* of nodes of $T_{act}$ is a *light* cluster if (1) its size fits into the range $I(n):= [\sqrt{n}, 2\sqrt{n}]$, (2) it is either a maximal subtree in $T_{act}$ or a collection of maximal subtrees having the same parent, and (3) $T_{act}$ does not contain any heavy nodes.^6^ In case $T_{act}$ is of size smaller than $\sqrt{n}$, we drop the first condition and still call $K = V_{act}$ a light cluster.

Given the definition above, we present Algorithm 1 that shows how to find such a cluster.

**Algorithm 1:**
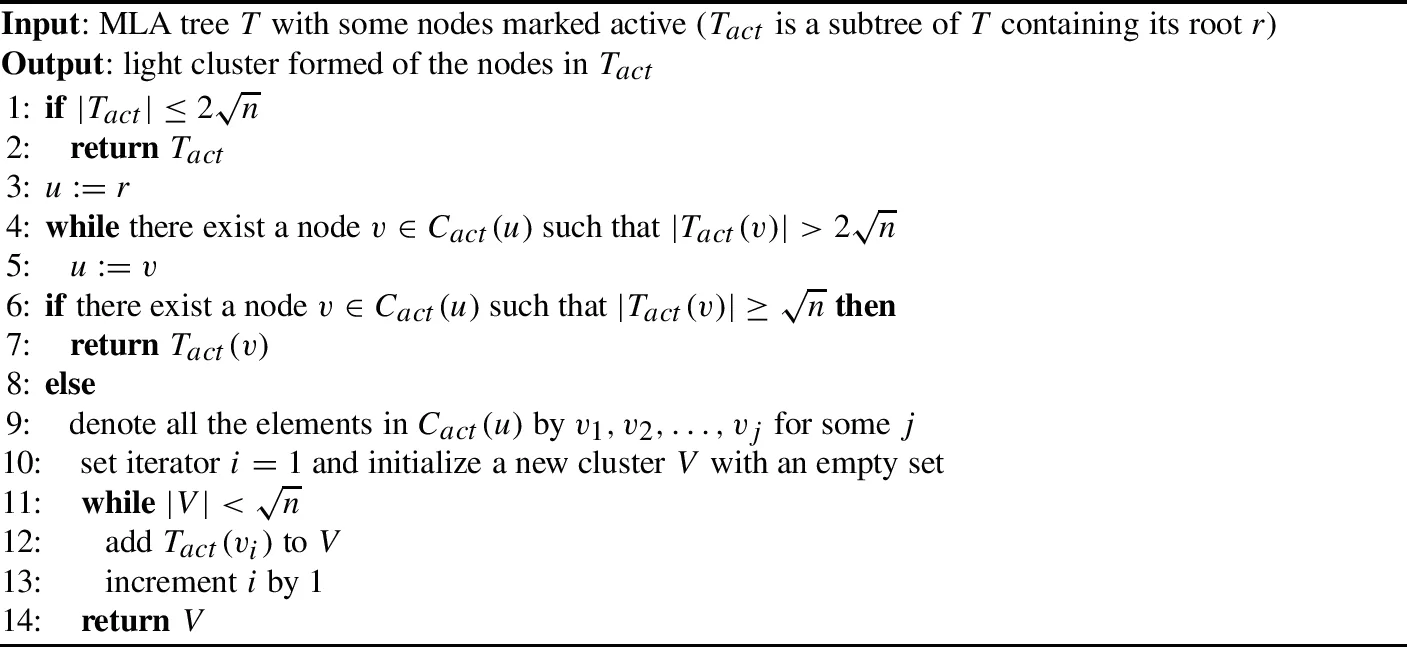
MLA Light Cluster Search

#### Proposition 3.5

If there are no heavy nodes in $T_{act}$ (see condition (3) in Definition 3.4), then Algorithm 1 finds a light cluster in $T_{act}$.

#### Proof

We first check whether $|T_{act}| \le 2\sqrt{n}$ (line 1). If so, we return the whole $T_{act}$, which matches the final case of Definition 3.4. Otherwise, we set u:=r (line 3) and repeatedly move to a child *v* whose subtree $T_{act}(v)$ has size $> 2\sqrt{n}$ (lines 4–5). The loop stops once no such child exists.

At this moment, two properties hold: (1) $$|T_{act}(u)| > 2\sqrt{n}$$, as we either remained at the root or descended only through nodes with subtrees of size $$> 2\sqrt{n}$$; (2) every child of *u* has subtree size $$\le 2\sqrt{n}$$, otherwise the loop would continue. Next, in line 6, we check whether some child *v* satisfies $$|T_{act}(v)| \ge \sqrt{n}$$. If so, we return $$T_{act}(v)$$ as a light cluster. Otherwise (line 8), we iterate over the children $$v_i$$ of *u* (line 11), adding their subtrees to a set *V* until $$|V| \ge \sqrt{n}$$, and return *V*. Since $$|T_{act}(u)| > 2\sqrt{n}$$, we must eventually reach the $$\sqrt{n}$$ threshold. Moreover, just before adding the last subtree $T^{\prime }$, we had $$|V| < \sqrt{n}$$, and because $$|T'| < \sqrt{n}$$, the resulting cluster has size $$< 2\sqrt{n}$$. □

**Main algorithm.**

Before we describe the partitioning algorithm, let us introduce a helper function. We define method $\texttt {cluster}(V)$ to group all the elements of *V* together and include them as a new cluster in the partition. Let us also emphasize that after this call, all the elements in *V* become inactive. With the above notation, we can formalize our approach as presented in Algorithm 2.

**Algorithm 2:**
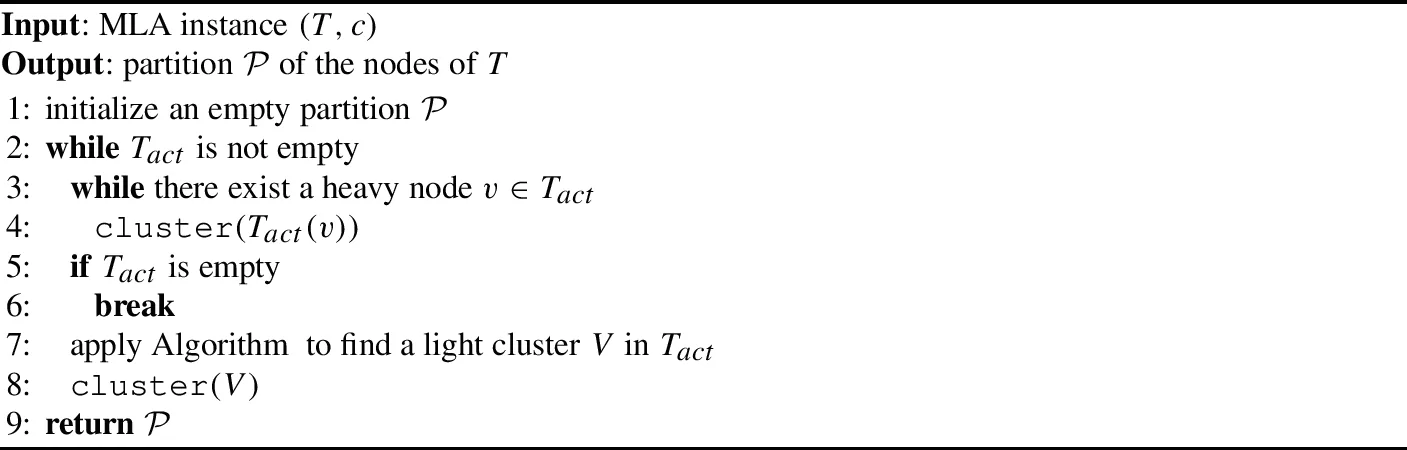
MLA Partitioning Algorithm

As mentioned in the first part of this section, the main partitioning algorithm runs heavy and light cluster searches in a loop. In the first step, it iteratively finds the heavy clusters in the tree $T_{act}$ determined by the already created partition (lines 3 to 4). Then, if tree *T* is not yet partitioned (condition in line 5 does not hold), it goes to the second step that finds one light cluster and adds it to the partition (lines 7 to 8). After this point, it goes to the initial step and loops.

Let us emphasize that during the whole partitioning procedure, the set $T_{act}$ of all active elements in *T* forms a proper subtree containing the root *r* of *T*. Indeed, in the beginning, $T_{act} = T$ and all the cluster calls truncate one or more maximal subtrees from $T_{act}$. Now, given Algorithm 2, we go back to proving the properties of light clusters.

#### Proposition 3.6

Let *T* be an MLA tree rooted at some node *r* and let $\mathcal {P}$ be the partition of nodes of *T* created by Algorithm 2. We denote all the light clusters in $\mathcal {P}$ by $P_{\ell ,1}, P_{\ell ,2}, \ldots , P_{\ell ,t}$ and require them to be listed in the creation order. Then, it holds that there are at most $\sqrt{n} + 1$ clusters $P_{\ell ,i}$.

#### Proof

Notice that by the definition, the only light cluster that can have a size smaller than $\sqrt{n}$ is the one containing the root *r*. Thus, all the light clusters created before, i.e., at least t-1 of them, have the size at least $\sqrt{n}$. Since there are *n* nodes in tree *T*, we get that there are at most $n/\sqrt{n} = \sqrt{n}$ such clusters. Thus, $t \le \sqrt{n} + 1$, which concludes the proof. □

In the remainder of this section, we refer to clusters created in lines 2, 7 of Algorithm 1, i.e., the ones that consist of a single subtree, as the light clusters of type I. We call the light clusters consisting of forests (created in line 14) the light clusters of type II. We prove that the cost function *c*, defined as for any $V \subseteq U, c(V) = \sum _{v \in V} c(v)$, satisfies the following properties.

#### Proposition 3.7

Let (*T*, *c*) be an MLA instance and let $\mathcal {P}$ be the partition obtained on it by Algorithm 2. Take any light cluster *K* in $\mathcal {P}$ and denote by $r_K$ the root of *K* if it is a cluster of type I. Otherwise, if *K* is a cluster of type II, we use $r_K$ to denote the parent node of the forest *K*. Then it holds that $c(R(r_K)) \ge c(K)$.

#### Proof

Assume first that *K* is of type I. Let *w* be the node in *K* that has the highest cost. By Definition 3.4, we know that $|K| \le 2\sqrt{n}$. Hence, by an averaging argument, we have $c(w) \ge c(K) / (2\sqrt{n})$, which implies $2\sqrt{n} \cdot c(w) \ge c(K)$. Notice that it implies $c(T(w) \cap K) < 4\sqrt{n} \cdot c(w)$. Now, assume by contradiction that $c(R(r_K)) < c(K)$. Then, if we split the path from *w* to *r* into two parts by cutting it on the node $r_K$, we get $c(R(w)) = c(R(w) \cap K) + c(R_{o}(r_K))$. Since $c(R(w) \cap K) \le c(K)$ and $c(R(r_K)) \le c(K)$ by our assumption, we get that $c(R(w)) \le 2c(K) \le 2 \cdot 2\sqrt{n} \cdot c(w) = 4\sqrt{n} \cdot c(w)$. However, this means that *w* is a heavy cluster root, which contradicts the initial assumption. Thus, it holds that $c(R(r_K)) \ge c(K)$. For a type II cluster, we have $c(R(w)) = c(R(w) \cap K) + c(R(r_K))$, and the same inequalities follow. □

#### Corollary 3.8

Let us subsume the notation and the conditions of Proposition 3.7. Then, it holds that $f(K) \le 2f(r_K)$.

#### Proof

Notice that *f*(*K*) consists of the cost of *K* and the cost of the path connecting it to the root *r* of *T* (to be precise, for the type I cluster we exclude $r_K$ from this path, as we already count its cost in the cluster). Thus, the following holds

$$ f(K) ~\le ~ c(K) + c(R(r_K)) \le c(R(r_K)) + c(R(r_K)) = 2 c(R(r_K)) = 2 f(r_K), $$

where the second inequality is implied by Proposition 3.7, and the last equality is by the definition of *f*. □

Given the above, we can prove the main theorem of this section.

#### Theorem 3.9

For any MLA service function *f*, there exists a disjoint service function *g* that $O(\sqrt{n})$-approximates *f*. It can be found in time polynomial w.r.t. the MLA instance defining *f*.

#### Proof

Let (*T*, *c*) be the MLA instance that defines *f*, and let *U* be the set of nodes in *T*. The idea is to prove that the partition $\mathcal {P} = \{P_1, P_2, \ldots , P_k\}$ generated on *T* by Algorithm 2 induces a set function $g(V)=\sum _{i\in [k]} f(P_i) \cdot 1\!\!1\left\{ P_i \cap V \ne \emptyset \right\}$ on all subsets V⊆U that is an $O(\sqrt{n})$-approximation to *f*. The function *g* is a disjoint service function by design.

For this purpose, we need to show that $$\max _{V \subseteq U} g(V) / f(V)$$ is of order at most $$\sqrt{n}$$. Let us note that in our case, *f*(*V*) is just the cost of the minimal subtree connecting *V* to the root. Thus, for any subset $V^{\prime }$ of *V* it holds that $f\left(V^{\prime }\right)\leq f\left(V\right)$.

Let V⊆U be any subset of nodes and let $P_{h,1}$, …, $P_{h,s}$ and $P_{\ell ,1}, P_{\ell ,2}, \ldots , P_{\ell ,t}$ be the lists of all the heavy and light clusters that intersect *V*, respectively. We also denote the roots of the heavy clusters by $v_{h,1}$, …, $v_{h,s}$, respectively, and the set containing them by $V_h$. Similarly, we use the convention from Proposition 3.7 to define roots of light clusters. For $P_{\ell ,i}$, we denote its root by $r_{\ell ,i}$.

By Proposition 3.3, it holds that $\sum _{i=1}^s f(P_{h,i}) \le 8\sqrt{n} \cdot f(V_h)$. Moreover, since *V* intersects all these heavy clusters, it either contains their roots or some nodes that are their descendants. Thus, the minimal subtree connecting *V* to the root *r* contains the minimal subtree connecting $V_h$ to the root *r*. Hence,

2 $$\begin{aligned} \sum _{i=1}^s f(P_{h,i}) \le 8\sqrt{n} \cdot f(V_h) \le 8\sqrt{n} \cdot f(V). \end{aligned}$$

Now, for each light cluster $P_{\ell ,i}$, we notice that since *V* intersects it, the minimal tree connecting *V* to *r* contains the path from $r_{\ell ,i}$ to *r*. Thus, $f(V) \ge f(r_{\ell ,i})$ and by Corollary 3.8 (using its notation), we get that

3 $$\begin{aligned} f(P_{\ell ,i}) \le 2f(r_K) \le 2f(V) \end{aligned}$$

for each $\ell \in [t]$. Note that $g(V) = \sum _{K \in \mathcal {P}: V \cap K \not = \emptyset } f(K)$. Combining inequalities 2 and 3, we obtain that

$$\begin{aligned} \begin{aligned} \frac{g(V)}{f(V)} = \frac{\sum _{K \in \mathcal {P}: V \cap K \not = \emptyset } f(K)}{f(V)}&= \frac{\sum _{i=1}^s f(P_{h,i}) + \sum _{i=1}^t f(P_{\ell ,i})}{f(V)}\\&\le \frac{8\sqrt{n} \cdot f(V) + \sum _{i=1}^t 2f(V)}{f(V)} \\&\le \frac{8\sqrt{n} \cdot f(V) + 2(\sqrt{n} + 1) \cdot f(V)}{f(V)} = 10\sqrt{n} + 2, \end{aligned} \end{aligned}$$

with the last inequality implied by Proposition 3.6. This concludes the proof that *g* is an $O(\sqrt{n})$-approximation to *f*.

Finally, it is easy to observe that the algorithm runs in polynomial time, as a simple dynamic structure over *T* (storing *c*(*T*(*v*)), *c*(*R*(*v*)), and |*T*(*v*)|) allows all necessary updates during clustering to be performed in linear time in *n*.

□

Thus, by Lemma 1.1, we get Theorem 1.2. The result of Theorem 3.9 is tight:

#### Proposition 3.10

There exists an MLA instance (*T*, *c*) with *n* nodes, such that for every partition $\mathcal {P} = \{P_1,\ldots ,P_k\}$ of *T* for some *k*, there exists a non-empty set S⊆T such that

$$\frac{\sum _{i\in [k]} f(P_i) \cdot 1\!\!1\left\{ S\cap P_i\ne \emptyset \right\} }{f(S)}= \Omega (\sqrt{n}) .$$

#### Proof

For simplicity, we assume that $\sqrt{n}$ is an integer. The argument extends to general *n* through straightforward rounding. Consider the tree *T* with a root *r* and n-1 children of *r* denoted by $v_1,\ldots ,v_{n-1}$. The cost *c* is such that $c(r)=\sqrt{n}$, while for all i∈[n-1], $c(v_i)=1$. Now, consider any partition $\mathcal {P} = \{P_1,\ldots ,P_k\}$. If $k>\sqrt{n}$, then consider a set *S* that intersects each $P_i$ exactly once. Thus, $f(S) \le \sqrt{n} + k \le 2k$, while $\sum _{i\in [k]} f(P_i) \cdot 1\!\!1\left\{ S\cap P_i\ne \emptyset \right\} \ge k \cdot \sqrt{n}$, which proves this case. Else ($k \le \sqrt{n}$), consider a set *S* that intersects $P_i$ once if and only if $|P_i| ~>~ \sqrt{n}/2$ (otherwise does not intersect at all). It holds that $f(S) \le 2\sqrt{n}$ because one has $\sqrt{n}$ from *r* and $\sqrt{n}$ intersections with $P_i$’s in the worst case, while

$$\sum _{i\in [k]} f(P_i) \cdot 1\!\!1\left\{ S\cap P_i\ne \emptyset \right\} \ge n-\sum _{i\in [k]} |P_i|\cdot 1\!\!1\left\{ S\cap P_i = \emptyset \right\} \ge n- k \sqrt{n}/2 \ge n/2, $$

which concludes the proof. □

## Weighted Symmetric Subadditive Joint Replenishment

In this section, we study Weighted Symmetric Subadditive JRP. We have a set *U* of *n* request types with weights $w(\{j\}) = w_j$ for each j∈U. Let *f* be the set function over *U*: In this setting, we have that the service cost of a set *S* only depends on the total weight of the elements belonging to *S*, as opposed to the identity of those elements. Formally, f(S)=f(w(S)), where function *f* is now intended as a monotone non-decreasing subadditive function of weights of a set with f(0)=0, and for every two weights *x*, *y*, it holds that f(x+y)≤f(x)+f(y). For brevity, we call these functions *weighted symmetric subadditive*. Our goal is to show that for every weighted symmetric subadditive service function *f* on *U*, there exists a partition of *U* into sets $S_1,\ldots ,S_k$ for some *k*, such that the disjoint service function $g: 2^U \rightarrow \mathbb {R}_{\ge 0}$ defined by this partition where $g(S) = \sum _{i=1}^k f(S_i)\cdot 1\!\!1[S\cap S_i \ne \emptyset ]$ satisfies $g(S) \le O(\sqrt{n})f(S)$ for every S⊆U.

We begin, in Section 4.1, by analyzing a special case of unweighted symmetric subadditive service costs. Namely, where the weight of each element is 1, and thus, w(S)=|S|: These functions are simply referred to as *symmetric subadditive*. We achieve a tight $\Theta (\sqrt{n})$-stretch with a simple partitioning algorithm (partition into $\sqrt{n}$ sets of size $\sqrt{n}$ each), and this serves as a warm-up to the weighted symmetric subadditive case presented in Section 4.2, where we also achieve a tight $\Theta (\sqrt{n})$-stretch.

### Symmetric Subadditive JRP

We first consider symmetric subadditive service functions. Observe that these functions are symmetric (i.e., $f\left(S\right)=f\left(S^{\prime }\right)$ for all sets $S,S^{\prime }\subseteq U$ such that $\left|S|=\right|S^{\prime }|$). For convenience, for a cardinality $$0\le s\le |U|$$, we use *f*(*s*) as the value of sets of size *s*. We show that for symmetric subadditive *f*, one can construct a disjoint service function *g* that $$O(\sqrt{n})$$-approximates *f*. We then show that $$O(\sqrt{n})$$ is tight even in the special case of *f* being a symmetric unweighted set cover function. This provides an alternative, simpler proof for the lower bound on USC of [35]. We first state the following simple but useful observation.

#### Observation 4.1

For all symmetric subadditive functions $f: \mathbb {R}_{+} \rightarrow \mathbb {R}_{+}$, and all y≥x>0, it holds that $f(y)/f(x) \le \lceil y/x \rceil$.

#### Proof

Let $k = \lceil y/x \rceil$. Then, $f(y) \le f(kx) \le f(x) + f((k-1)x) \le f(x) +\ldots +f(x) = k \cdot f(x)$. The first inequality is by monotonicity, and the second and third by subadditivity. □

#### Lemma 4.2

For every symmetric subadditive service function *f*, there exists a disjoint service function *g* that $O(\sqrt{n})$-approximates it.

#### Proof

Let us consider an arbitrary symmetric subadditive service function *f* on request types *U*. Let *g* be the disjoint service function that induces an arbitrary partition of the elements of *U* into sets $\{X_1, \ldots , X_k\}$, where $k = \lceil \sqrt{n}\rceil$, each of cardinality $|X_i| \le \lceil \sqrt{n}\rceil$ (such a partition always exists). We now bound the following fraction for every S⊆U:

$$\begin{aligned} \frac{\sum _{i \in [k]} f(X_i) \cdot 1\!\!1\left\{ S \cap X_i \ne \emptyset \right\} }{f(S)}&\le \frac{\sum _{i \in [k]} f(\lceil \sqrt{n} \rceil ) \cdot 1\!\!1\left\{ S \cap X_i \ne \emptyset \right\} }{f\left( \sum _{i \in [k]} 1\!\!1\left\{ S \cap X_i \ne \emptyset \right\} \right) } \\&\le \left( \sum _{i \in [k]} 1\!\!1\left\{ S \cap X_i \ne \emptyset \right\} \right) \cdot \Bigg \lceil \frac{\lceil \sqrt{n} \rceil }{\sum _{i \in [k]} 1\!\!1\left\{ S \cap X_i \ne \emptyset \right\} }\Bigg \rceil \\&\le 2\lceil \sqrt{n} \rceil . \end{aligned}$$

The first inequality is because $|X_i| \le \lceil \sqrt{n}\rceil$ and from the fact that, since $X_i$’s are disjoint, the size of *S* is at least the number of non-empty intersections with sets $X_i$’s. The second inequality follows from Observation 4.1, and the third inequality follows since $\lceil \frac{a}{b}\rceil \le 2 \cdot \frac{a}{b}$, for every $\frac{a}{b} \ge \frac{1}{2}$, and the denominator $\sum _{i \in [k]} 1\!\!1\left\{ S \cap X_i \ne \emptyset \right\} \le \sqrt{n} + 1$. □

Thus, by Lemma 1.1 and Lemma 4.2, the following holds:

#### Theorem 4.3

There exists a deterministic $O(\sqrt{n})$-competitive algorithm for the Non-Clairvoyant Symmetric Subadditive Joint Replenishment problem.

We complement the above result by giving a tight instance:

#### Theorem 4.4

There exists a symmetric subadditive service function such that every disjoint service function is an $\Omega (\sqrt{n})$-approximation of it.

#### Proof

Let *U* be the set of request types. For simplicity of the proof, we assume that n=|U| has an integer square root. Let us consider the service function $f(S) = \Big \lceil \frac{|S|}{\sqrt{n}}\Big \rceil$, which is symmetric and subadditive, let *g* be any disjoint service function, let $\mathcal {S}$ be the collection of disjoint sets $X_i$’s that *g* generates, and let *k* be the number of clusters in the partition $\mathcal {S}$.

Consider some X⊆U that intersects each $X_i$ exactly once, and this means that |X|=k. We now analyze the cost of this induced partition on *X*:

$$ \frac{\sum _{i=1}^k f(X_i)}{f(X)} = \frac{\sum _{i=1}^k \Big \lceil \frac{|X_i|}{\sqrt{n}}\Big \rceil }{\Big \lceil \frac{k}{\sqrt{n}}\Big \rceil } \ge \frac{\max \{k,\sqrt{n}\}}{\Big \lceil \frac{k}{\sqrt{n}}\Big \rceil } , $$

where the inequality holds since the it is a sum of *k* terms where each is at least 1, and since the sets $X_1,\ldots ,X_k$ cover *U*, thus $\sum _{i=1}^k |X_i| = n$.

Now, if $k \le \sqrt{n}$ then

$$ \frac{\max \{k,\sqrt{n}\}}{\Big \lceil \frac{k}{\sqrt{n}}\Big \rceil } = \sqrt{n}.$$

Otherwise, $\frac{k}{\sqrt{n}} >1$, thus $\Big \lceil \frac{k}{\sqrt{n}}\Big \rceil \le 2 \frac{k}{\sqrt{n}}$, which implies that

$$ \frac{\max \{k,\sqrt{n}\}}{\Big \lceil \frac{k}{\sqrt{n}}\Big \rceil } \ge \frac{k}{2k/\sqrt{n}} = \frac{\sqrt{n}}{2},$$

which concludes the proof. □

### Weighted Symmetric Subadditive JRP

We now relax the assumption of w(S)=|S| and provide a $O(\sqrt{n})$-approximation for every weighted subadditive function.

We begin with some facts about weighted subadditive and symmetric concave functions. Every symmetric concave function is the pointwise infimum of a set of affine functions, and can be approximated by a set of affine functions with geometrically decreasing slopes. The next lemma combines this fact with the fact that every weighted subadditive function can be approximated by a symmetric concave function.

#### Lemma 4.5

Let $g:\{0,1\ldots ,W\}\rightarrow \mathbb {R}_{\ge 0 }$ be a monotone non-decreasing subadditive function. Then, there exists a finite set of $p = \lceil \log (W)\rceil$ affine functions $\{g_1,\ldots ,g_p\}$ where $g_i(x) = \sigma _i + x\cdot \delta _i$, and the function $\hat{g}$ defined by $\hat{g}(x) = \min _i g_i(x)$ satisfies that for every $x\in \{0,\ldots ,W\}$, it holds that:

$$\begin{aligned} \frac{g(x)}{4} \le \hat{g}(x) \le g(x). \end{aligned}$$

#### Proof

By [28], we know that there exists a concave function $g^\prime :\{0,\ldots ,W\}\rightarrow \mathbb {R}_{\ge 0}$ that approximates *g* within a factor of 2. Now, for every $i=2,\ldots ,\lceil \log (W)\rceil$ consider the affine function $g^\prime _i: \{0,\ldots ,W\}\rightarrow \mathbb {R}_{\ge 0}$ that interpolates between $(2^{i-1},g^\prime (2^{i-1}))$ and $(2^{i},g^\prime (2^{i}))$, and $g_1'(x)$ that interpolates between $(0,g^\prime (0))$ and $(1,g^\prime (1))$. Note that, by concavity of $g^\prime$, we have the following properties:

1. For i≥2 and $x \in \{2^{i-1}, \ldots , 2^i\}$ or i=1 and $x\in \{0,1\}$, it holds that $i \in \arg \min _{j=1,\ldots ,p} g_j(x)$.
2. For i≥2 and $x \in \{2^{i-1}, \ldots , 2^i\}$ or i=1 and $x\in \{0,1\}$, it holds that $g_i(x) \le g^\prime (x)$.

Therefore, for every $x \in \{0,\ldots ,W\}$, it holds that

$$\begin{aligned} g^\prime (x)&\le g^\prime (2^{\lceil \log (x)\rceil }) \le 2g^\prime (2^{\lfloor \log (x)\rfloor }) = 2g^\prime _{\lfloor \log (x)\rfloor }(2^{\lfloor \log (x)\rfloor }) \\&= 2\min _{i=1,\ldots ,p} g^\prime _i(2^{\lfloor \log (x)\rfloor }) \le 2\min _{i=1,\ldots ,p} g^\prime _i(x), \end{aligned}$$

where the second equality is by property 1. Together with property 2 (implying $\min _{i=1,\ldots ,p} g^\prime _i(x) \le g^\prime (x)$ for every $x\in \{0,\ldots ,W\}$), we have that

$$\begin{aligned} \frac{g^\prime (x)}{2} \le \hat{g}(x) \le g^\prime (x). \end{aligned}$$

Combining with that $g(x)/2\le g'(x)\le g(x)$, concludes the proof of the lemma. □

In [34, Lemmas 3.1 and 3.2], the authors present an algorithm that reduces the set of affine functions (“pipes” in their terminology) such that the coefficients and slopes satisfy the conditions of the lemma while losing a factor of 2. This applied to the set of affine functions $g^\prime _i$ of Lemma 4.5 yields:

#### Corollary 4.6

Let $g:\{0,1\ldots ,W\}\rightarrow \mathbb {R}_{\ge 0 }$ be a monotone non-decreasing subadditive function. Then, there exists a finite set of $p \le \lceil \log (W)\rceil$ affine functions $\{g_1,\ldots ,g_p\}$ where $g_i(x) = \sigma _i + x\cdot \delta _i$, and the function $\hat{g}$ defined by $\hat{g}(x) = \min _i g_i(x)$ such that $\sigma _{i+1} > 2\sigma _i$ and $\delta _{i+1}< \delta _i / 2$ for every i<p satisfies that for every $x\in \{0,\ldots ,W\}$, it holds that:

$$\begin{aligned} \frac{g(x)}{16} \le \hat{g}(x) \le g(x). \end{aligned}$$

Henceforth, we will denote by W=w(U), and assume that *f* is defined on $\{0,\ldots ,W\}$, and is a pointwise minimum of *p* affine functions $g_1, \ldots , g_p$ where $g_i(x) = \sigma _i + x \cdot \delta _i$ and the $\sigma _i$s and $\delta _i$s satisfy the properties stated in the corollary. Proving the theorem for *f* that satisfies the condition proves the same (with additional loss of a factor of 16) for general symmetric subadditive functions.

The following lemma will be useful to lower bound *f*(*w*(*S*)) using the largest weight in *S*.

#### Lemma 4.7

For every $k \in \{2,\ldots ,p\}$, if $x \ge \frac{\sigma _k}{\delta _{k-1}}$ , then $f(x) \ge \sigma _k$.

#### Proof

Recall that $f(x) = \min _{1 \le i \le p} \sigma _i + x\delta _i$. For i<k, we have $\sigma _i + x\delta _i\ge x \delta _{k-1} \ge \sigma _k$. For i≥k, we have $\sigma _i + x \delta _i \ge \sigma _k$. Thus, $f(x) \ge \sigma _k$. □

Henceforth, for brevity, we write *f*(*S*) to mean *f*(*w*(*S*)), for an arbitrary set *S*. In the following, we frequently use the fact that for any set *H*, $f(H) = \min _{1 \le i' \le p} \sigma _{i'} + w(H)\delta _{i'} \le \sigma _i + w(H)\delta _i$ for every *i*.

**High-Level Overview.** Determining the optimal size for each part involves striking a delicate balance. Larger sizes enable us to leverage the subadditivity of *f* but excessively large sizes incur higher costs for smaller sets. We demonstrate that selecting sets of size $O(\sqrt{n})$ is the optimal tradeoff in worst-case scenarios. Notably, this partition remains effective across all symmetric subadditive functions simultaneously.

Extending this approach to the general case of weighted symmetric subadditive functions involves categorizing elements into weight classes based on powers of 2, ensuring approximate size equivalence, and then partitioning into sets of size $\sqrt{n}$. However, this approach risks generating an excessive number of sets. To address this issue, we devise a partitioning strategy that accommodates light-weight elements first. Then, for heavier-weight elements, we further partition by a factor of 2, provided it is feasible, to achieve a refined division.

**Notational Details.** Let *S* be a set chosen by an adversary, unknown to us. Suppose that $f(S) = \min _{1 \le i \le p} \sigma _i + w(S)\delta _i = \sigma _\ell + w(S)\delta _\ell$ for some ℓ. The idea is to construct a partition such that some of the parts that intersect *S* can be charged to $\sigma _\ell$, and the remaining parts that intersect *S* can be charged to $w(S)\delta _\ell$. Towards this end, we first classify each type *j* as follows. We say that type *j* is *eligible* for *class* $2 \le k \le p$ if $w_j \ge \frac{\sigma _k}{\delta _{k-1}}$. All types are eligible for class 1. Define the *class* of type *j* to be the largest class it is eligible for and $X_k$ to be the set of class-*k* types.

Next, we partition $X_k$ into heavy and light types. The light part $Z_k$ contains all types $j \in X_k$ with $w_j\delta _k \le \sigma _k/\sqrt{n}$. Since $Z_k$ is light, $f(Z_k) \le \sigma _k + w(Z_k) \delta _k \le O(\sqrt{n})\sigma _k$. Also, if $S \cap X_k \ne \emptyset$, then Lemma 4.7 implies that $f(S) \ge \sigma _k$. We can then use the fact that $\sigma _k$’s are geometric to show that the total value of the parts $Z_k$ that intersect *S* is at most $O(\sqrt{n})f(S)$.

Now, consider the heavy types in $X_k$, i.e. those types *j* with $w_j\delta _k > \sigma _k/\sqrt{n}$. We further partition these types according to their weights in powers of 2. Let $R_{k,i} = \{j \in X_k \setminus Z_k: w_j \in [2^i, 2^{i+1})\}$. For each weight class *i*, we greedily partition $R_{k,i}$ into as many parts of size $\lceil \sqrt{n} \rceil$ as we can. This produces a collection $F_{k,i}$ of parts of size $\lceil \sqrt{n} \rceil$ and at most one leftover part $G_{k,i}$ of size less than $\sqrt{n}$. We say that a part is *nice* if it belongs to $F_{k,i}$ and the part $G_{k,i}$ is a *leftover* part.

Observe that there are at most $\lceil \sqrt{n} \rceil$ nice parts, each of size at most $\lceil \sqrt{n} \rceil$ and contains types of roughly the same weight. Thus, we can use a similar argument as in the unweighted case to show that the total value of the nice parts that intersect *S* is at most $O(\sqrt{n})f(S)$. For the leftover parts, we charge the parts $G_{k,i}$ that intersect *S* with k<ℓ to $w(S)\delta _\ell$ and those with $k \ge \ell$ to $\sigma _\ell$.

**Algorithm 3:**
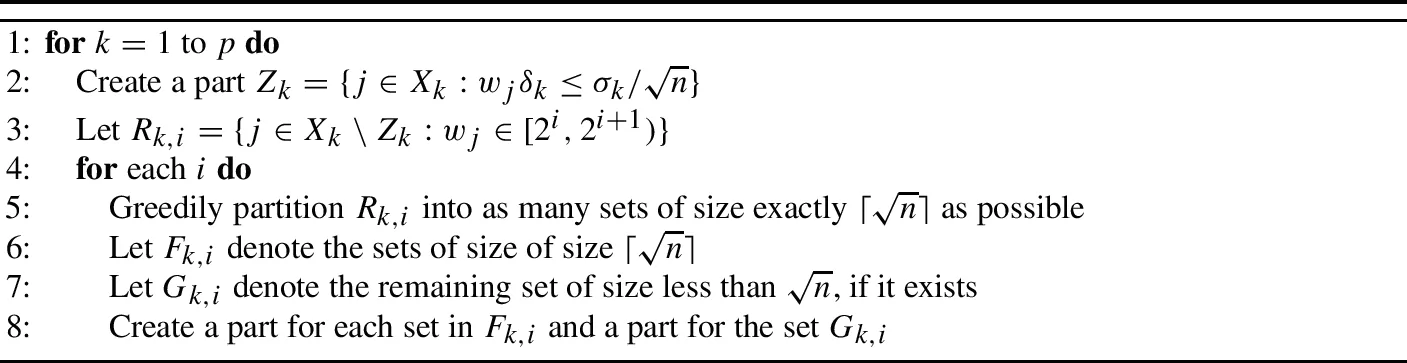
Weighted Symmetric Subadditive Partitioning Algorithm

We now give the detailed analysis below.

#### Theorem 4.8

For any weighted symmetric subadditive service function *f*, there exists a disjoint service function *g* that $O(\sqrt{n})$-approximates *f*. It can be found in time polynomial w.r.t. the weights defining *f*.

#### Proof

Let *S* be an arbitrary set and suppose $f(S) = \min _{1 \le i \le p} \sigma _i + w(S)\delta _i = \sigma _\ell + w(S)\delta _\ell$ for some ℓ. We now decompose *w*(*S*) using the partition produced by our algorithm. In particular, we have

$$\begin{aligned} f(S) = \sigma _\ell + \left( \sum _k w(Z_k \cap S) + \sum _{k, i} \sum _{T \in F_{k,i}} w(T \cap S) + \sum _{k, i} w(G_{k,i} \cap S)\right) \cdot \delta _\ell . \end{aligned}$$

Define $F'_{k, i}$ as the subset of parts in $F_{k, i}$ that intersects with *S*. We now show that the algorithm pays at most $O(\sqrt{n})f(S)$. In other words, we will prove that the total value of the parts that intersect *S* are upper bounded as follows:

$$\begin{aligned} \sum _{k : Z_k \cap S \ne \emptyset } f(Z_k) + \sum _{k, i} \sum _{T \in F'_{k,i}} f(T) + \sum _{k, i : G_{k,i} \cap S \ne \emptyset } f(G_{k,i})\le O(\sqrt{n})f(S). \end{aligned}$$

We begin by bounding $\sum _{k: Z_k \cap S \ne \emptyset } f(Z_k)$. Let $k_{\operatorname {max}}$ be the largest *k* such that $Z_k \cap S \ne \emptyset$. (If none exists, then we do not need to bound this term.) First, assume that $k_{\operatorname {max}}\ge 2$. We have that $w(S)\cdot \delta _{k_{\operatorname {max}}-1} \ge w(Z_k \cap S)\cdot \delta _{k_{\operatorname {max}}-1} \ge \sigma _{k_{\operatorname {max}}}$. Thus, Lemma 4.7 implies that $f(S) \ge \sigma _{k_{\operatorname {max}}}$. For $k_{\operatorname {max}}= 1$, for some *i* it holds that $f(S) = \sigma _i + w(S) \delta _i \ge \sigma _1 = \sigma _{k_{\operatorname {max}}}$. On the other hand,

$$\begin{aligned} \sum _{k : Z_k \cap S \ne \emptyset } f(Z_k) \le \sum _{k : Z_k \cap S \ne \emptyset } O(\sqrt{n}) \sigma _k \le O(\sqrt{n}) \sigma _{k_{\operatorname {max}}} \le O(\sqrt{n})f(S), \end{aligned}$$

where the first inequality follows directly from the definition of $Z_k$ in line 2 of Algorithm 3 and given that there are at most *n* elements in $Z_k$; the second inequality is since the $\sigma _k$’s are geometrically increasing.

Next, we bound $\sum _{k, i} \sum _{T \in F'_{k,i}} f(T)$. Since every set $T \in F'_{k,i}$ has size $\lceil \sqrt{n} \rceil$, we have $\sum _{k,i} |F'_{k,i}| \le \sqrt{n}$. Moreover, every j∈T has $w_j \in [2^i, 2^{i+1})$, so $w(T) \le O(\sqrt{n}) w(T \cap S)$. Indeed, *T* can have at most $\lceil \sqrt{n} \rceil$ times more elements than T∩S (whenever the intersection is non-empty), and by the earlier condition all elements of *T* have the same weight up to a factor of two. Thus, we have

$$\begin{aligned} \sum _{k, i} \sum _{T \in F'_{k,i}} f(T)&\le \sum _{k, i} \sum _{T \in F'_{k,i}} \sigma _\ell + w(T)\delta _\ell \\&\le \sum _{k,i} |F'_{k,i}| \sigma _\ell + O(\sqrt{n})\sum _{k, i} \sum _{T \in F'_{k,i}}w(T \cap S) \delta _\ell \\&\le O(\sqrt{n}) \left( \sigma _\ell + \sum _{k, i} \sum _{T \in F'_{k,i}} w(T \cap S) \delta _\ell \right) \le O(\sqrt{n}) f(S). \end{aligned}$$

where the last inequality follows from the fact that all $T \in F^\prime _{k,i}$ are disjoint so we have that $w(S) \ge \sum _{k, i} \sum _{T \in F'_{k,i}} w(T \cap S)$.

We now turn to bounding $\sum _{k, i: G_{k,i} \cap S \ne \emptyset } f(G_{k,i})$. Consider a set $G_{k,i}$ that intersects *S* for $\ell \le k \le p$. Since $G_{k,i}$ is a subset of $X_k \setminus Z_k$ and is at most of size $\sqrt{n}$, we have that

$$f(G_{k,i}) \le \sigma _k + w(G_{k,i})\delta _k \le O(\sqrt{n}) w(G_{k,i} \cap S) \delta _k.$$

Since $\delta _k \le \delta _\ell$, we get that

$$\sum _{k\ge \ell } \sum _{i : G_{k,i} \cap S \ne \emptyset } f(G_{k,i}) \le \sum _{k\ge \ell } \sum _{i : G_{k,i} \cap S \ne \emptyset } O(\sqrt{n}) w(G_{k,i} \cap S)\delta _\ell \le O(\sqrt{n}) f(S).$$

Finally, when ℓ=1, the argument is complete. Let us now consider the case when ℓ>1. Consider a set $G_{k,i}$ that intersects *S* for $1 \le k < \ell$. We have that $f(G_{k,i}) \le \sigma _k + w(G_{k,i})\delta _k \le O(\sqrt{n}) 2^{i+1} \delta _k$. Moreover, since every $j \in X_k$ has $w_j \delta _k < \sigma _{k+1}$, we have that

$$\sum _{k< \ell } \sum _{i : G_{k,i} \cap S \ne \emptyset } f(G_{k,i}) \le O(\sqrt{n}) \sum _{k < \ell } \sigma _{k+1} \le O(\sqrt{n}) \sigma _\ell \le O(\sqrt{n})f(S).$$

Finally, it is not hard to see that, by design, Algorithm 3 can be implemented in polynomial time in the logarithm of the total weight, log(w(U)). This concludes the proof. □

Theorem 1.3 follows by Lemma 1.1 together with Theorem 4.8.

## Tight Instances against Previous Algorithms

### An $\Omega (\sqrt{n\log n})$ Tight Instance for the Algorithm of [35]

#### Proposition 5.1

There exists a weighted set cover instance for which the Universal Set Cover algorithm of [35] has stretch $\Omega (\sqrt{n \log n})$.

#### Proof

The algorithm of [35] works as follows: while the set *U* of elements *e* for which a(e) is undefined is non-empty, pick the set *S* that minimizes $\frac{c(S)}{\sqrt{|S \cap U|}}$ and for all $e \in S \cap U$, define a(e)=S.

The high-level idea is that [35]’s analysis uses the Cauchy-Schwarz inequality and the tight instance is created by looking at when the Cauchy-Schwarz inequality is tight.

Consider the following set system where we have sets $S, S_1, \ldots , S_k$ for some *k* that we will choose later. The set *S* contains *k* elements and set $S_i$ contains $\left\lfloor \frac{k}{k - (i-1)}\right\rfloor$ elements. The sets also satisfy that $|S \cap S_i| = 1$ and $S_i \cap S_j = \emptyset$ for $1 \le i < j \le k$. Moreover, the sets $S_i$ form a partition of all the *n* elements. The costs of the sets are: c(S)=1+ε (for an arbitrarily small ε>0), $c(S_i) = \frac{\sqrt{|S_i|}}{\sqrt{k-(i-1)}}$.

We now claim that in the *i*-th iteration, the algorithm chooses $S_i$. First observe that for $1 \le i < j \le k$, we have

$$\begin{aligned} \frac{c(S_i)}{\sqrt{|S_i|}} < \frac{c(S_j)}{\sqrt{|S_j|}}. \end{aligned}$$

Thus, it suffices to show that in each iteration *i*, the algorithm chooses $S_i$ over *S*. We do this by induction on *i*. When i=1, we have that

$$\begin{aligned} \frac{c(S_1)}{\sqrt{|S_1|}} = \frac{1}{\sqrt{k}} ~<~ \frac{c(S)}{\sqrt{|S|}}. \end{aligned}$$

Now consider i>1. By induction, we have that |S∩U|=k-(i-1) and $S_i \cap U = S_i$ (the latter is because the only set that intersects $S_i$ is *S*). Thus, we also have

$$\begin{aligned} \frac{c(S_i)}{\sqrt{|S_i|}} = \frac{1}{\sqrt{k - (i-1)}} ~<~ \frac{c(S)}{\sqrt{|S \cap U|}}. \end{aligned}$$

We conclude that in each iteration *i*, the algorithm chooses $S_i$.

Thus, the competitive ratio of the algorithm is at least $(\sum _{i=1}^k c(S_i))/c(S) = \sum _{i=1}^k c(S_i)/(1+\varepsilon )$ since c(S)=1+ε. We have that

4 $$\begin{aligned} \frac{\sum _{i=1}^k c(S_i)}{1+\varepsilon } = \frac{1}{1+\varepsilon } \cdot \sum _{i=1}^k \frac{\sqrt{\left\lfloor \frac{k}{k - (i-1)}\right\rfloor }}{\sqrt{k-(i-1)}} = \Omega (\sqrt{k} \log k). \end{aligned}$$

It now remains to maximize *k*. The constraint on *k* is that $\sum _{i =1}^k |S_i| = n$ since $S_i$’s are disjoint. Now, $\sum _{i=1}^k |S_i| = \sum _{i=1}^k \left\lfloor \frac{k}{k - (i-1)}\right\rfloor = \Theta (k \log k)$. Thus, setting $k = \Theta (n/\log n)$ satisfies the constraint on *k*. Plugging this into (4) yields the claim. □

### An $\Omega (\sqrt{n\log n})$ Tight Instance for the Algorithm of [44]

**Fig. 3:**
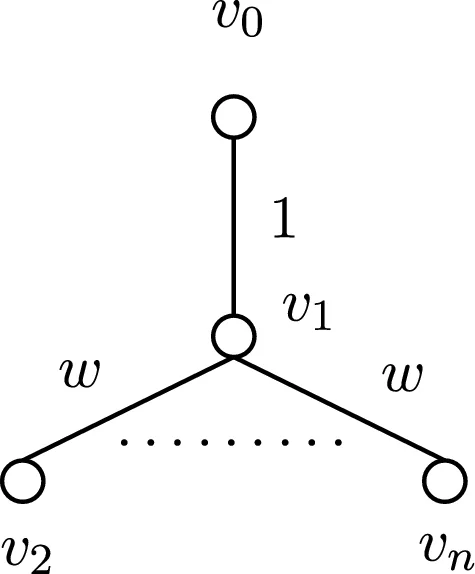
Tight instance for [44], where $w = \frac{\sqrt{n \log n} - 1}{n-1}$.

We complement the $O(\sqrt{n})$-stretch achieved by Algorithm 2 and Algorithm 3 with a subadditive JRP instance such that the algorithm of [44] (Algorithm 2) must suffer a stretch of at least $\Omega (\sqrt{n\log n})$. Note that the instance we present in Figure 3 is both an MLA instance and a weighted symmetric subadditive one. This shows that for the specific case of MLA and weighted symmetric subadditive functions, not only is our algorithm optimal, but also that Touitou’s algorithm cannot achieve the same guarantee. At a high level, whenever Touitou’s algorithm decides to serve some requests, it issues up to two services (lines 9 and 12). One of them serves a subset of requests *R* for which delay and service costs are the same. At the same time, a second service with a budget of up to $\sqrt{n \log n} \cdot c(R)$ can be issued to serve some pending requests in advance. The following example is one where the optimal algorithm rarely issues this second service.

#### Proposition 5.2

There exists an instance for which the algorithm of [44] has stretch $\Omega (\sqrt{n \log n})$. Moreover, this is an MLA and a weighted symmetric subadditive instance.

#### Proof

Let us consider the JRP tree *T* in Figure 3, where $w = \frac{\sqrt{n \log n} - 1}{n-1}$ and the delay cost functions on the nodes read

$$ d_i(t) = {\left\{ \begin{array}{ll} 2t, {\text { if }} i = 1 \\ \varepsilon t, {\text { if }} i \ge 2 \end{array}\right. }, $$

for $\varepsilon =\frac{1}{n^2w}$. At each time step, there are *n* requests arriving on tree *T*, one per node.

Let τ be the length of the sequence. Consider the algorithm that serves requests at $v_1$ immediately once they arrive, and once every $s =nw\le \tau$ times, it serves all of the requests at nodes $v_1, \ldots , v_n$. The algorithm has a service cost equal to s-1+(n-1)w+1=s+(n-1)w every *s* consecutive times. Overall, we have service cost equal to $\frac{\tau }{s} \cdot (s + (n-1)w)$ to serve $v_1, \ldots , v_n$. Moreover, the delay cost of the algorithm is 0 for requests at $v_1$, and for each $i\in \{2,\ldots ,n\}$, within each *s* times the delay cost is $\frac{\varepsilon s^2}{2}$, thus the overall delay cost is $(n-1)\frac{\tau }{s} \cdot \frac{\varepsilon s^2}{2} = \frac{(n-1)\tau \varepsilon s}{2}$. Overall,

$$ OPT (\tau ) \le \tau \left( 1 + \frac{(n-1)w}{s} + \frac{(n-1)\varepsilon s}{2}\right) \le 3\tau . $$

Algorithm 2 in [44] (whose cost is referred to as ALG from now on) serves a request arriving at $v_1$ (line 9) as soon as its accumulated delay equals its service cost (this is when the UponCritical event occurs). Once a request at $v_1$ arrives, the algorithm waits until the time elapsed *t* is such that 2t=1 to serve it. That is, when the *j*-th request located at $v_1$ arrives, the algorithm serves it at time $t_j = j + \frac{1}{2}$. Right after, it issues a second service (line 12) to serve all other requests at $v_2, \ldots , v_n$. Overall, the algorithm pays $ALG (\tau ) = \tau \cdot (1 + (n-1)w) = \tau \sqrt{n \log n}$.

Hence,

$$\frac{ALG (\tau )}{OPT (\tau )} \ge \frac{\sqrt{n \log n}}{3},$$

for all $\tau \ge 1$. To conclude, the fact that the instance in Figure 3 is an MLA one comes directly from the fact that it is a depth 2 tree. Moreover, observe that no matter how we choose S⊆V, f(S)=f(w(S)), and thus the instance in Figure 3 is also a weighted symmetric subadditive instance. □

## Data Availability

No datasets were generated or analysed during the current study.

## Footnotes

1 A set function over a ground set *U* is *subadditive* if $f(A) + f(B) \ge f(A \cup B)$ for every A,B⊆U.

2 Observe that this captures the case of deadlines by delay costs that are 0 up to the deadline and then increase very rapidly such that the delay cost is much higher than the serving cost before the arrival of the next request. Thus, it is always better to serve them at the deadline.

3 We assume without loss of generality that $d_q(a_q) = 0$, i.e., serving a request immediately on arrival incurs no delay cost.

4 Several other classes within the submodular class have been considered (e.g. additive, unit-demand, gross-substitutes).

5 We use the term symmetric submodular to indicate functions that are (monotone) concave in the size of the set.

6 This third condition is for analysis purposes only and the property giving the name to *light* clusters.
